# The Alpha-Synuclein RT-QuIC Products Generated by the Olfactory Mucosa of Patients with Parkinson’s Disease and Multiple System Atrophy Induce Inflammatory Responses in SH-SY5Y Cells

**DOI:** 10.3390/cells11010087

**Published:** 2021-12-28

**Authors:** Chiara Maria Giulia De Luca, Alessandra Consonni, Federico Angelo Cazzaniga, Edoardo Bistaffa, Giuseppe Bufano, Giorgia Quitarrini, Luigi Celauro, Giuseppe Legname, Roberto Eleopra, Fulvio Baggi, Giorgio Giaccone, Fabio Moda

**Affiliations:** 1 Division of Neurology 5, Neuropathology, Fondazione IRCCS Istituto Neurologico Carlo Besta, 20133 Milan, Italy; chiara.deluca@istituto-besta.it (C.M.G.D.L.); federico.cazzaniga@istituto-besta.it (F.A.C.); edoardo.bistaffa@istituto-besta.it (E.B.); g.bufano@campus.unimib.it (G.B.); giorgia.quitarrini01@universitadipavia.it (G.Q.); giorgio.giaccone@istituto-besta.it (G.G.); 2 Laboratory of Prion Biology, Department of Neuroscience, Scuola Internazionale Superiore Di Studi Avanzati (SISSA), 34136 Trieste, Italy; lcelauro@sissa.it (L.C.); legname@sissa.it (G.L.); 3 Division of Neurology 4, Neuroimmunology and Neuromuscular Diseases, Fondazione IRCCS Istituto Neurologico Carlo Besta, 20133 Milan, Italy; alessandra.consonni@istituto-besta.it (A.C.); fulvio.baggi@istituto-besta.it (F.B.); 4 Division of Neurology 1, Parkinson and Movement Disorders, Fondazione IRCCS Istituto Neurologico Carlo Besta, 20133 Milan, Italy; roberto.eleopra@istituto-besta.it

**Keywords:** RT-QuIC, olfactory mucosa, α-synuclein, Parkinson’s disease, multiple system atrophy, strains

## Abstract

Parkinson’s disease (PD) and multiple system atrophy (MSA) are caused by two distinct strains of disease-associated α-synuclein (αSyn^D^). Recently, we have shown that olfactory mucosa (OM) samples of patients with PD and MSA can seed the aggregation of recombinant α-synuclein by means of Real-Time Quaking-Induced Conversion (αSyn_RT-QuIC). Remarkably, the biochemical and morphological properties of the final α-synuclein aggregates significantly differed between PD and MSA seeded samples. Here, these aggregates were given to neuron-like differentiated SH-SY5Y cells and distinct inflammatory responses were observed. To deepen whether the morphological features of α-synuclein aggregates were responsible for this variable SH-SY5Y inflammatory response, we generated three biochemically and morphologically distinct α-synuclein aggregates starting from recombinant α-synuclein that were used to seed αSyn_RT-QuIC reaction; the final reaction products were used to stimulate SH-SY5Y cells. Our study showed that, in contrast to OM samples of PD and MSA patients, the artificial aggregates did not transfer their distinctive features to the αSyn_RT-QuIC products and the latter induced analogous inflammatory responses in cells. Thus, the natural composition of the αSyn^D^ strains but also other specific factors in OM tissue can substantially modulate the biochemical, morphological and inflammatory features of the αSyn_RT-QuIC products.

## 1. Introduction

Parkinson’s disease (PD) and multiple system atrophy (MSA) are neurodegenerative disorders belonging to a group of pathologies named α-synucleinopathies [1,2]. These diseases are characterized by the accumulation of misfolded α-synuclein in neurons or glial cells of PD and MSA patients, respectively [3]. It is known that they are caused by different conformational states of α-synuclein (αSyn), also referred to as α-synuclein strains, or αSyn^D^ [4,5,6]. αSyn^D^ promotes conformational templating of normally folded αSyn resulting in αSyn^D^ spreading in specific brain areas with the formation of insoluble aggregates [7,8,9,10,11].

Besides αSyn^D^ accumulation, specific neuroinflammatory responses are known to contribute to the neurodegenerative processes. Remarkably, toll-like receptor (TLR) signaling is a major pathway mediating inflammation, and TLR2 expression is known to be increased in PD [12,13]. TLR2 is involved in the induction of pro-inflammatory mediators by triggering glial activation, autophagy-mediated neuronal αSyn^D^ accumulation and clearance, and pathogenic neuron-to-neuron and neuron-to-glia αSyn^D^ transmission [14]. Moreover, activation of neuronal TLR2 may induce an inflammatory response, resulting in microglia activation, release of inflammatory mediators, as well as the production of mitochondrial reactive oxygen species [15]. Whether different αSyn^D^ strains could trigger distinct inflammatory pathways is still under debate and the contribution of TLRs, particularly TLR2, in this response has yet to be elucidated. Cytokines such as IL6, TNFα, and IFNγ have been reported to be increased in the serum of PD and MSA patients [16,17], but their role on neurons and glial cells to sustain the neuroinflammatory process has to be better investigated. Moreover, in the context of PD and MSA, it has been demonstrated that misfolded αSyn may be released from neurons and can seed intracellular αSyn aggregation while promoting cytokines release and the shift to a pro-inflammatory environment [18]. αSyn^D^ is phagocytosed by microglia and triggers its activation, thus further contributing to the neurodegenerative process [19].

Thanks to the development of the Seed Amplification Assays (SAAs), traces of αSyn^D^ were found in peripheral tissues of patients with α-synucleinopathies, including cerebrospinal fluid (CSF) [20,21,22,23,24,25], skin [26,27,28,29], submandibular gland [26,30,31] and olfactory mucosa [25,32,33,34]. Recently, we have optimized the Real-Time Quaking-Induced Conversion (RT-QuIC) assay for detecting αSyn^D^ in OM samples of patients with PD and MSA [33,34]. The αSyn_RT-QuIC exploited the ability of αSyn^D^ (also referred to as seed) present in OM tissues to template the conformational conversion of recombinant αSyn (rec-αSyn, used as RT-QuIC reaction substrate) which then aggregated to form αSyn amyloid fibrils [20,24,26]. Notably, the biochemical and morphological properties of the RT-QuIC generated αSyn fibrils were significantly different between PD and MSA and enabled diseases discrimination [33]. In 2020, similar findings were published by Shahnawaz et al. who showed that the RT-QuIC analysis of CSF from patients with PD and MSA led to the formation of αSyn fibrils useful to readily distinguish between pathologies [35]. It is therefore conceivable that αSyn^D^ may influence the morphological properties of rec-αSyn aggregates that are formed during the αSyn_RT-QuIC reaction, and this could confer them also specific inflammatory properties.

For this reason, we have exposed neuron-like differentiated SH-SY5Y cells to αSyn fibrils generated by RT-QuIC analysis of OM-MSA and OM-PD to evaluate whether distinct inflammatory responses were induced. This cellular model has been extensively used to investigate several prion-like properties of αSyn^D^, including cell-to-cell αSyn^D^ propagation, the formation of intracellular aggregates and related toxic effects, especially after stimulation with recombinant preformed αSyn fibrils (PFF) [36,37,38,39].

To better understand whether the inflammatory properties observed in SH-SY5Y cells were associated with specific biochemical or morphological features of the RT-QuIC products, we have generated three distinct αSyn aggregates (named αSv1, αSv2 and αSv3) using rec-αSyn. These samples were either challenged in SH-SY5Y cells, or subjected to αSyn_RT-QuIC to test whether and to what extent they could imprint their specific features to the reaction substrate. We are aware of the fact that αSv1, αSv2 and αSv3 lacked several post-translational modifications (e.g., phosphorylation, nitration, ubiquitination) [40] that might influence their seeding activity, including the possibility to transmit specific morphological properties to the reaction products. For this reason, we have performed additional studies using the αSyn aggregates formed in SH-SY5Y cells stimulated with αSv1, αSv2 or αSv3 whose structure and composition could better resemble that of the natural αSyn^D^ strains.

Our findings suggest a link between the aberrant structures of αSyn and the inflammatory pathways activated in SH-SY5Y cells. However, contrary to what has been observed in the case of OM-MSA and OM-PD samples, the artificial aggregates made up of rec-αSyn and those generated in SH-SY5Y cells did not transmit specific properties to the αSyn_RT-QuIC products. Therefore, the natural αSyn^D^ strains responsible for MSA and PD (likely in combination with other still unknown factors present in the OM tissue) possess unique features which are barely reproducible using in vitro models.

## 2. Materials and Methods

*Expression and purification of recombinant human wild-type α-synuclein.* Pet11a plasmid with the sequence encoding for the recombinant human wild-type α-synuclein (rec-αSyn) was expressed in BL21 (DE3) E. coli strain (Stratagene, San Diego, CA, USA). One hundred milliliters of overnight culture was inoculated into M9 minimal medium (1X M9 salts, 2 mM MgSO_4_, 0.1 mM CaCl_2_, 0.4% glucose) complemented with 100 µg/mL ampicillin and growth at 37 °C under shaking until 0.6 O.D. measured at 600 nm. The induction of the construct was obtained by growing cells with 0.6 mM isopropyl b-D galactopyranoside (IPTG) for 5 h. Protein extraction was performed as described [41]. Briefly, the cell pellet was resuspended in osmotic shock buffer (30 mM Tris-HCl, 2 mM EDTA, 40% sucrose, pH 7.2) and boiled for 10 min while stirring. After two subsequent precipitation steps with 35% and 55% of ammonium sulfate, the protein was dialyzed in 20 mM Tris-HCl pH 8 and purified by anion exchange chromatography (HiTrap Q FF column, cytiva, Washington, MA, USA). The elution was obtained by a NaCl 0–500 mM linear gradient. The purity of the protein was confirmed by SDS-PAGE and the fractions containing rec-αSyn were dialyzed into water, quantified by measuring the absorbance at 280 nm, lyophilized (FreeZone 2.5 Freeze Dry System, Labconco, Kansas City, MO, USA) and stored at −80 °C.

*Generation of α-synuclein aggregates with different morphological features.* Rec-αSyn was thawed and diluted to a final concentration of 21 μM in three different aggregation buffers: 1. H_2_O (αSv1); 2. 5 mM Tris and 100 mM NaCl (αSv2); 3. 5 mM Tris (αSv3). All reagents used to prepare the reaction mixes were filtered through a 0.22 µm filter before use. Reactions were performed in sixfold in a black 96-well optical flat bottom plate (Thermo Fisher Scientific, Waltham, MA, USA) using the Fluoroskan Ascent microplate reader (Thermo Fisher Scientific, Waltham, MA, USA). One hundred microliters of reaction mix was added to each well, together with a glass bead (3-mm, Sigma). The plate was sealed with a sealing film (Thermo Fisher Scientific, Waltham, MA, USA) and subjected to continuous shaking (600 rpm, single orbital) at 37 °C. An additional well was prepared for each buffer and was supplemented with 10 μM Thioflavin-T (ThT) to monitor rec-αSyn aggregation. Fluorescence intensities, expressed as arbitrary units (AU), were taken every 60 min using 450 ± 10 nm (excitation) and 480 ± 10 nm (emission) wavelengths, with a bottom read. Once reached the fluorescence plateau, rec-αSyn aggregates were collected and characterized by means of biochemical (Western blot, dot blot, dye-binding assay) and morphological (transmission electron microscopy, TEM) analyses.

*Collection and preparation of olfactory mucosa samples.* Olfactory mucosa (OM) samples were collected before the COVID-19 pandemic with non-invasive procedures from extensively characterized patients with a clinical diagnosis of probable PD (*n* = 2) or MSA-P (*n* = 2) or healthy control (HC, *n* = 1). The nasal cavity was treated with a topical anesthetic (Ecocain, Molteni Dental, Milan, Italy) for 10 min and the OM were collected between the septum and the middle turbinate by gently brushing with a cotton swab (FLOQSwabs^TM^ Copan Italia, Brescia, Italy), as previously described [33]. After collection, cotton swabs were immersed in saline solution, the olfactory cells were separated by vigorous vortexing and finally pelleted at 800× *g* for 20 min at 4 °C. Approximately 6 μg of the pellets were collected with inoculating loops and suspended in 50 µL of PBS for αSyn_RT-QuIC analysis. The study and its ethical aspects were approved by the ethical committee of Fondazione IRCCS Istituto Neurologico Carlo Besta. All of the participants provided written informed consent before OM collection and analysis.

*αSyn_RT-QuIC analysis of olfactory mucosa samples.* The αSyn_RT-QuIC reaction mix was composed by 14 μM rec-αSyn, 40 mM PBS (pH 8.0), 170 mM NaCl and 10 μM ThT. All the reagents were filtered through a 0.22 µm filter before use. Two microliters of OM sample prepared as previously described was added to 98 µL of the reaction mix. All samples were analyzed in triplicate in a black 96-well optical flat bottom plate (Thermo Fisher Scientific, Waltham, MA, USA) preloaded with a glass bead (3-mm, Sigma, Saint Louis, MO, USA) per well. The plate was sealed with a sealing film (Thermo Fisher Scientific, Waltham, MA, USA) and subjected to cycles of shaking (1 min at 600 rpm, single orbital) and incubation (14 min at 42 °C) using the Fluoroskan Ascent microplate reader (Thermo Fisher Scientific, Waltham, MA, USA). Fluorescence intensities, expressed as AU, were taken every 15 min using 450 ± 10 nm (excitation) and 480 ± 10 nm (emission) wavelengths, with a bottom read. Final reaction products were named RQ-MSA1, RQ-MSA2, RQ-PD1, RQ-PD2 and RQ-CTRL.

*αSyn_RT-QuIC analysis of αSv1, αSv2, and αSv3.* αSv1, αSv2, and αSv3 were serially diluted (volume/volume) in PBS (1.5 µg, 1.5 ng, 1.5 pg, 1.5 fg, and 1.5 ag) and 5 µL of pure or diluted samples was added to 95 µL of αSyn_RT-QuIC reaction mix that was performed as described in “αSyn_RT-QuIC analysis of olfactory mucosa samples”. Final reaction products were named RQ-αSv1, RQ-αSv2 and RQ-αSv3. As control, unseeded αSyn_RT-QuIC reactions (RQ-no seed) were performed.

*Dye-binding assay of αSv1, αSv2, and αSv3.* To perform the dye-binding assay, αSv1, αSv2, and αSv3 were prepared without ThT. Samples were then diluted to a final concentration of 5 μM in PBS and divided in 8 aliquots that were incubated with 8 different dyes (at room temperature, in the dark): 10 μM ThT, 10 μM 4,4′-bis-1-anilinonaphthalene-8-sulfonate (Bis-ANS), 5 μM Congo red, Amytracker 480 (1:800 in H_2_O), Amytracker 520 (1:800 in H_2_O), Amytracker 540 (1:800 in H_2_O), Amytracker 630 (1:800 in H_2_O) and Amytracker 680 (1:800 in H_2_O). After 30 min, samples were added to a black 384-well optical flat bottom plate (Thermo Fisher Scientific, Waltham, MA, USA). This latter was sealed with a sealing film (Thermo Fisher Scientific, Waltham, MA, USA) and inserted in a ClarioSTAR microplate reader (BMG Labtech, Ortenberg, Germany). The fluorescence values were recorded using the appropriate wavelengths: 448/482 nm exc/emi for ThT, 400/505 nm exc/emi for Bis-ANS, 544/620 exc/emi for Congo red, 430/480 nm exc/emi for Amytracker 480, 470/520 nm exc/emi for Amytracker 520, 470/540 nm exc/emi for Amytracker 540, 510/630 nm exc/emi for Amytracker 630 and 540/680 nm exc/emi for Amytracker 680.

*Cell culture and stimulation.* Undifferentiated SH-SY5Y neuroblastoma cells were maintained in Dulbecco’s Modified Eagle Medium (DMEM) with 2 mM L-glutamine, 1X penicillin/streptomycin, and supplemented with 10% Fetal Calf Serum (FCS) at 37 °C, 5% CO_2_. Differentiation of SH-SY5Y cells into neuron-like cells was achieved by 10 μM trans-retinoic acid in DMEM 1% FCS, for 7 days, and seeded on 24 well-plates for molecular biology analysis, lysate preparation (250,000 cells/well), and for immunofluorescence analysis (70,000 cells/well). Cells were exposed to RQ-MSA1, RQ-MSA2, RQ-PD1, RQ-PD2, αSv1, αSv2, αSv3, RQ-αSv1, RQ-αSv2, RQ-αSv3 and related controls (final concentration 2.5 µM) for 24 h. For lysate preparation, SH-SY5Y cells were detached with PBS by scraping. Supernatants were centrifuged at 10,000 rpm for 5 min and stored at −80 °C.

*RT-qPCR analysis.* cDNA was synthesized from total RNA (TRIzol, Thermo Fisher Scientific, Waltham, MA, USA) using random hexamers, and reverse transcriptase (SuperScript VILO cDNA Synthesis Kit, Thermo Fisher Scientific, Waltham, MA, USA). Real-time quantitative PCR (RT-qPCR) for TLR2 (Hs02621280_s1), TLR6 (Hs01039989_s1), TRAF6 (Hs00939742_g1), IL6 (Hs00174131_m1), NLRP3 (Hs00918080_g1), SOD2 (Hs00167309_m1) expression was performed using Assays-on-Demand Gene Expression assay (Thermo Fisher Scientific, Waltham, MA, USA). GAPDH (Hs02758991_G1) was used as housekeeping endogenous gene. Target mRNA expression was calculated as mean 2-ΔCtx100 value, in which ΔCt is the difference between target and housekeeping Ct. Real-time PCR reactions were performed in duplicate using ViiA7 Real-Time PCR System (Thermo Fisher Scientific, Waltham, MA, USA), according to the manufacturer’s instructions.

*Confocal microscopy analysis.* Neuron-like SH-SY5Y cells, seeded on 13 mm coverslips and exposed to RQ-MSA1, RQ-MSA2, RQ-PD1, RQ-PD2, αSv1, αSv2, αSv3, RQ- αSv1, RQ-αSv2, RQ-αSv3 and related controls for 24 h, were fixed with 4% paraformaldehyde in PBS (pH 7.0), permeabilized with 0.5% Triton-X100 in PBS, and incubated for 1 h in PBS- 5%BSA 2% NGS (blocking solution). TLR2 expression was detected with the mouse monoclonal antibody anti-TLR2 (TL2.1, Invitrogen, Waltham, MA, USA) and α-synuclein was detected with the mouse monoclonal antibody 4D6 (Abcam, Cambridge, UK), followed by Alexa Fluor-555 donkey anti-mouse secondary antibody (Thermo Fisher Scientific). Alexa Fluor 488^®^ phalloidin (Thermo Fisher Scientific, Waltham, MA, USA) was used to stain F-actin (cytoskeleton). Nuclei were stained with 4′,6-diamidino-2-phenylindole (DAPI) (Thermo Fisher Scientific, Waltham, MA, USA). Isotype control antibodies were used as negative controls (non-specific background). Maximum projection images from 10-slice Z-stack (300 nm step size) were acquired via confocal microscopy (C1/TE2000-E microscope; Nikon, Tokyo, Japan) using 40× (NA 1.30) and 100× (NA 1.40) oil objectives; at least 5 adjacent image fields were analyzed. Parameters for image acquisition were not modified to allow the comparison of fluorescence intensity as a measure of relative quantification. Image analysis was performed with Image J and FIJI software [42].

*NO release analysis.* NO production was determined by measuring the accumulation of nitrite in the culture medium. Nitrite was assayed colorimetrically by a diazotization reaction using the Griess reagent, composed of a 1:1 mixture of 1% sulfanilamide in 5% orthophosphoric acid and 0.1% naphtylethylenediamine dihydrochloride in water. One hundred microliters of culture medium was mixed to 100 μL of Griess reagent in a 96-multiwell plate and the O.D. at 550 nm was measured within 10 min. The nitrite concentration in the samples was interpolated from a NaNO_2_ standard curve ranging from 0 to 100 μΜ. The limits of detection and quantification were 0.25 and 0.7 μM, respectively.

*Multiparametric assay.* A Bio-Plex Pro^TM^ Human Cytokine 27-plex Immunoassay 96-well kit (Bio-Rad Laboratory, Hercules, CA, USA) was used to measure the concentration of pro- and anti-inflammatory cytokines, chemokines and growth factors in cells stimulated with αSv1, αSv2 and αSv3. The immunoassay includes: gamma interferon (IFNγ), interleukin-1β (IL1β), IL1 receptor antagonist (IL1ra), interleukin-2 (IL2), IL4, IL6, IL9, IL15, IL17, tumor necrosis factor-alpha (TNFα), interferon gamma-induced protein 10 (IP10).

*αSyn_RT-QuIC analysis of SH-SY5Y cell lysates stimulated with RQ-MSA, RQ-PD, αSv1, αSv2 or αSv3.* SH-SY5Y cells stimulated with RQ-MSA, RQ-PD, αSv1, αSv2 or αSv3 were lysed as described in the section “Cell culture and stimulation” and analyzed by αSyn_RT-QuIC. Five microliters of each lysate, named CS-RQ-MSA, CS-RQ-PD, CS-αS1, CS-αS2 or CS-αS3, respectively, was added to 95 µL of αSyn_RT-QuIC reaction mix that was performed as described in αSyn_RT-QuIC analysis of olfactory mucosa samples. Final reaction products were named RQ-CS-RQ-MSA1, RQ-CS-RQ-MSA2, RQ-CS-RQ-PD1, RQ-CS-RQ-PD2, RQ-CS-αS1, RQ-CS-αS2 and RQ-CS-αS3.

*PK digestion assays.* Eight microliters of αSv1, αSv2 or αSv3 and all αSyn_RT-QuIC products were subjected to limited proteolytic digestion with Proteinase K (PK, Invitrogen) at the final concentration of 10 and 50 μg/mL, respectively. Digestions were performed under shaking (500 rpm), at 37 °C for 60 min. PK activity was stopped by the addition of LDS-PAGE loading buffer and boiling of the samples at 100 °C for 10 min.

*Western blot analysis.* Eight microliters of samples, either untreated or digested with PK, were loaded into 12% Bolt Bis-Tris Plus gels (Invitrogen), subjected to SDS-PAGE and transferred onto polyvinylidene difluoride (PVDF) membranes (Immobilon-P, Millipore, Burlington, MA, USA). Membranes were first incubated with paraformaldehyde (0.4% in PBS) for 30 min (under shaking) at room temperature and then blocked with non-fat dry milk (5% in TBS with 0.05% Tween-20, TBST) for 60 min (under shaking) at room temperature. Finally, membranes were immunoblotted with specific primary antibody directed against the N-terminal part of the protein (monoclonal AS08 358 Agrisera, Vännäs, Sweden: epitopes 1–15), overnight (under shaking) at 4 °C. After washing 3 times with TBST, membranes were incubated with the secondary antibody conjugated with horseradish peroxidase (Amersham donkey against rabbit IgG-HRP, diluted 1:2000 in TBST supplemented with 5% non-fat dry), subjected again to 3 washes with TBST and developed with chemiluminescent system (ECL Prime, cytiva). Reactions were visualized using a G:BOX Chemi Syngene system (Syngene, Bangalore, India).

*Dot blot analysis.* Two microliters of each αSyn aggregate or final αSyn_RT-QuIC products was diluted with 100 µL of PBS containing 0.1% Tween-20. Two microliters of the diluted samples were loaded onto nitrocellulose membranes (0.2 µm pore) and, after 30 min, the membranes were blocked with non-fat dry milk (5% in TBST) for 60 min (under shaking) at room temperature. Membranes were then incubated with a rabbit monoclonal αSyn filament-specific antibody (Abcam ab209538, MJFR-14-6-4-2, diluted 1:5000 in 5% BSA-PBS) for 60 min (under shaking) at room temperature. After washing 3 times with TBST, the membranes were incubated with the secondary antibody (Amersham donkey against rabbit IgG-HRP, diluted 1:10,000 in 5% BSA-PBS) for 60 min at room temperature. After 3 washes in TBST, membranes were developed with chemiluminescent system (ECL Prime) and reactions were visualized using a G:BOX Chemi Syngene system.

*Transmission electron microscopy analyses.* For transmission electron microscopy (TEM), all αSyn aggregates were properly diluted in water and 10 µL of the final dilutions was dropped onto 200-mesh Formvar-carbon coated nickel grids. After 30 min, the remaining drop was dried using filter paper and the samples were negatively stained with 25% Uranyl Acetate Replacement (UAR) for 10 min. Finally, the remaining staining solution was removed with filter paper and the grids were air-dried for 15 min. Samples were analyzed with a FEI Tecnai Spirit (FEI Company, Hillsboro, OR, USA), equipped with an Olimpus Megaview G2 camera.

*Statistical analyses.* Data related to cell analyses were expressed as the mean ± SD of two independent experiments. One-way ANOVA test, followed by Dunnett’s multiple comparison test, was used to evaluate statistical differences; *p*-values were corrected for multiple comparisons. *p* < 0.05 was considered statistically significant. GraphPad Prism v8.0 was used to represent αSyn_RT-QuIC kinetics, to elaborate data and to perform statistical analyses.

## 3. Results

### 3.1. αSyn_RT-QuIC Analysis of OM Samples and Biochemical Characterization of Reaction Products

**Figure 1:**
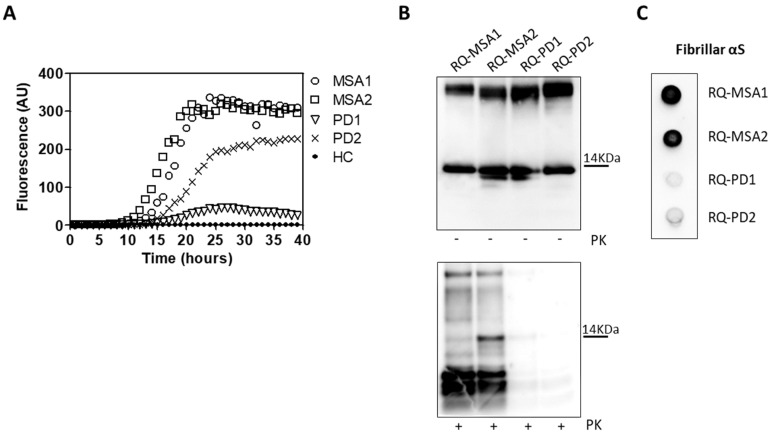
(**A**) αSyn_RT-QuIC analysis of OM-MSA and OM-PD samples. OM-MSA1, OM-MSA2, OM-PD1 and OM-PD2 induced a seeding activity while OM-HC did not. Curves represented in the graph were obtained by plotting the average fluorescence intensities of each sample against time. (**B**) Western blot analysis of untreated or PK digested αSyn_RT-QuIC products, collected at 40 h. RQ-MSA1, RQ-MSA2, RQ-PD1 and RQ-PD2 showed similar signal intensities before digestion. RQ-MSA1 and RQ-MSA2 were more resistant to PK digestion than RQ-PD1 and RQ-PD2. Blots were immunostained with the AS08 358 antibody. Number on the right indicates the molecular weight. (**C**) Dot blot analysis of αSyn_RT-QuIC products, collected at 40 h. Using the αSyn filament-specific MJFR antibody, RQ-MSA1 and RQ-MSA2 showed a more intense signal than RQ-PD1 and RQ-PD2.

OM collected from MSA and PD patients (OM-MSA1, OM-MSA2, OM-PD1 and OM-PD2) induced an efficient αSyn_RT-QuIC seeding activity while that of HC (OM-HC) did not (Figure 1A). Final reaction products were digested with PK and analyzed by Western blot using the AS08 358 antibody. As observed in our previous study [33], the αSyn_RT-QuIC products generated by OM-MSA (RQ-MSA1 and RQ-MSA2) were considerably more resistant to proteolytic digestion than those generated by OM-PD (RQ-PD1 and RQ-PD2) (Figure 1B). Dot blot analysis performed with the MJFR antibody, which recognizes fibrillary forms of α-synuclein, showed higher signal intensity in RQ-MSA1 and RQ-MSA2 samples than RQ-PD1 and RQ-PD2, thus suggesting that the aggregates possessed distinct morphological properties (Figure 1C).

### 3.2. Inflammatory Profile of Neuronal-like SH-SY5Y Cells Exposed to αSyn_RT-QuIC Products Generated by OM-MSA and OM-PD

**Figure 2:**
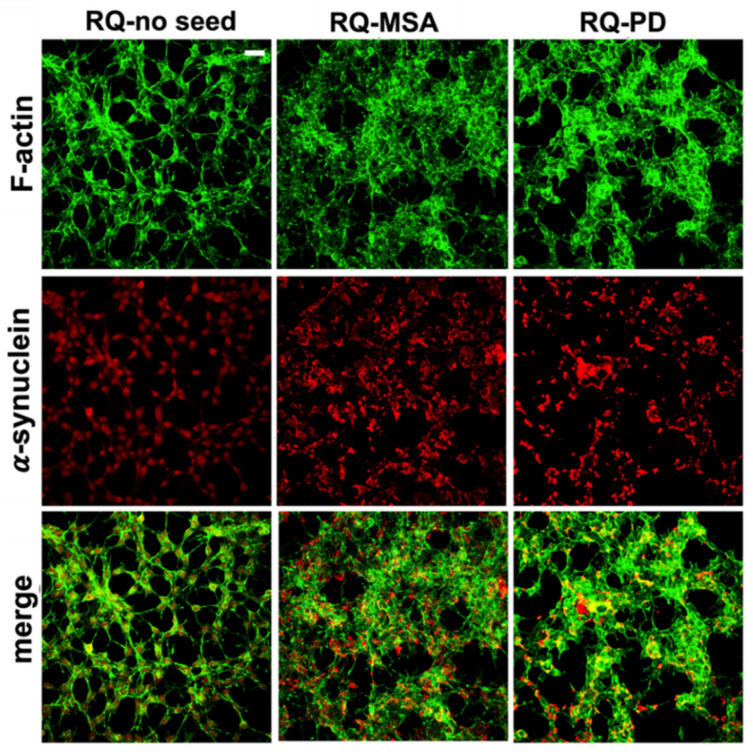
Intracellular αSyn aggregates in human differentiated neuroblastoma cells (SH-SY5Y) stimulated with RQ-MSA and RQ-PD. Representative pictures of SH-SY5Y cells incubated with RQ-MSA, RQ-PD (2.5 μM) or the unseeded αSyn_RT-QuIC reaction mix (RQ-no seed) were stained with mouse monoclonal antibody against αSyn (4D6), followed by Alexa Fluor-555 donkey anti-mouse secondary Abs (red) to show the presence of intracellular aggregates of αSyn. SH-SY5Y cells were counterstained with fluorescently-conjugated Alexa-Fluor 488-Phalloidin (green) to highlight the thin neurite-like cytoplasmic structures (cell-to-cell contacts) denoted by F-actin filaments. Scale bar = 25 µm.

Exposure of differentiated SH-SY5Y cells to RQ-MSA1, RQ-MSA2, RQ-PD1 and RQ-PD2 for 24 h induced intracellular αSyn aggregation. The results obtained from RQ-MSA1 and RQ-MSA2 were mediated and collectively named RQ-MSA while those obtained from RQ-PD1 and RQ-PD2 were mediated and collectively named RQ-PD. In particular, by immunofluorescence analysis, we have observed that cells cultured with RQ-MSA and RQ-PD showed the presence of clusters of αSyn aggregates mainly localized in the cytoplasm (Figure 2, red), whereas cells exposed to unseeded αSyn_RT-QuIC reaction mix (RQ-no seed) were characterized by a diffuse pattern of αSyn expression. The distribution and amount of αSyn deposits were similar between RQ-MSA and RQ-PD exposed SH-SY5Y cells (Figure 2, red) and no alterations in the structure of the cytoskeleton were observed, as confirmed by F-actin staining (Figure 2, green).

**Figure 3:**
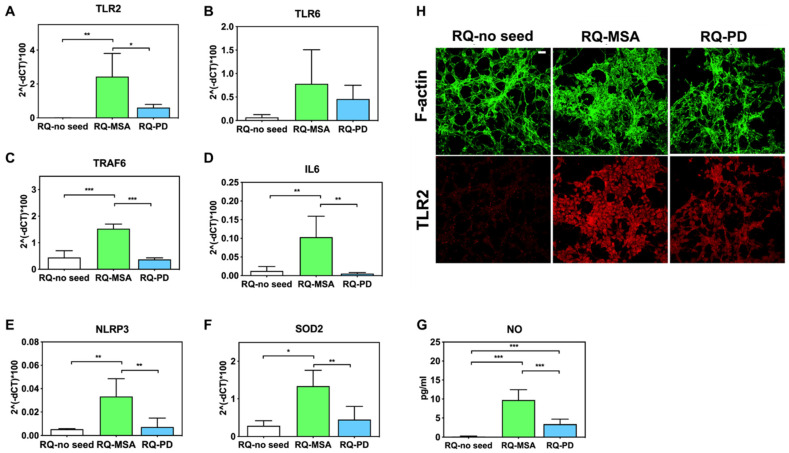
Analysis of inflammatory molecules in differentiated SH-SY5Y cells stimulated with RQ-MSA, RQ-PD and RQ-no seed. RT-qPCR analysis of TLR2 (**A**), TLR6 (**B**), TRAF6 (**C**), IL6 (**D**), NLRP3 (**E**), SOD2 (**F**) transcripts in SH-SY5Y exposed to RQ-MSA, RQ-PD (2.5 μM), and RQ-no seed for 24 h (mean ± SD). (**G**) NO release (mean ± SD) quantification through Griess’ reaction in the supernatants of cells stimulated with RQ-MSA, RQ-PD and RQ-no seed. Statistical significance was assessed by one-way ANOVA test with Dunnett’s multiple comparison test. Corrected *p*-values are reported in the results section. * *p* ≤ 0.05, ** *p* ≤ 0.01, *** *p* ≤ 0.001. (**H**) Confocal microscopy analysis showed increased TLR2 expression (red) in SH-SY5Y exposed to RQ-MSA and RQ-PD. Counterstaining with Alexa-Fluor 488-Phalloidin (green) was performed to monitor the cytoskeleton structure that appeared preserved in all experimental conditions. Scale bar = 25 µm.

Inflammatory effects were analyzed by means of RT-qPCR; SH-SY5Y cells exposed to RQ-PD and RQ-MSA showed an increase in transcription levels of inflammatory mediators, including TLR2, TLR6, TRAF6, IL6, NLRP3 inflammasome, and SOD2 (Figure 3). In particular, cells stimulated with RQ-MSA showed a significantly higher expression levels for TLR2 (RQ-MSA vs. RQ-no seed: *p* = 0.005; RQ-MSA vs. RQ-PD: *p* = 0.01) (Figure 3A), TRAF6 (RQ-MSA vs. RQ-no seed: *p* < 0.0001; RQ-MSA vs. RQ-PD: *p* < 0.0001) (Figure 3C), IL6 (RQ-MSA vs. RQ-no seed: *p* = 0.008; RQ-MSA vs. RQ-PD: *p* = 0.001) (Figure 3D), NLRP3 (RQ-MSA vs. RQ-no seed: *p* = 0.008; RQ-MSA vs. RQ-PD: *p* = 0.003) (Figure 3E), and SOD2 (RQ-MSA vs. RQ-no seed: *p* = 0.01; RQ-MSA vs. RQ-PD: *p* = 0.0042) (Figure 3F) than those stimulated with RQ-PD and RQ-no seed. Although upregulated, TLR6 (Figure 3B) expression levels did not reach statistical significance. Moreover, we have evaluated the levels of nitrites and nitrates (generally referred to as NO) in culture supernatants and found a significant increase of NO release in cells exposed to both RQ-MSA and RQ-PD compared to unseeded αSyn_RT-QuIC reaction mix (RQ-MSA vs. RQ-no seed: *p* < 0.0001; RQ-MSA vs. RQ-PD: *p* < 0.001; RQ-PD vs. RQ-no seed: *p* = 0.004) (Figure 3G). The increased TLR2 expression in RQ-MSA and RQ-PD stimulated cells was further confirmed by immunofluorescence analysis (Figure 3H).

### 3.3. Generation and Morphological Characterization of Recombinant α-Synuclein Aggregates

**Figure 4:**
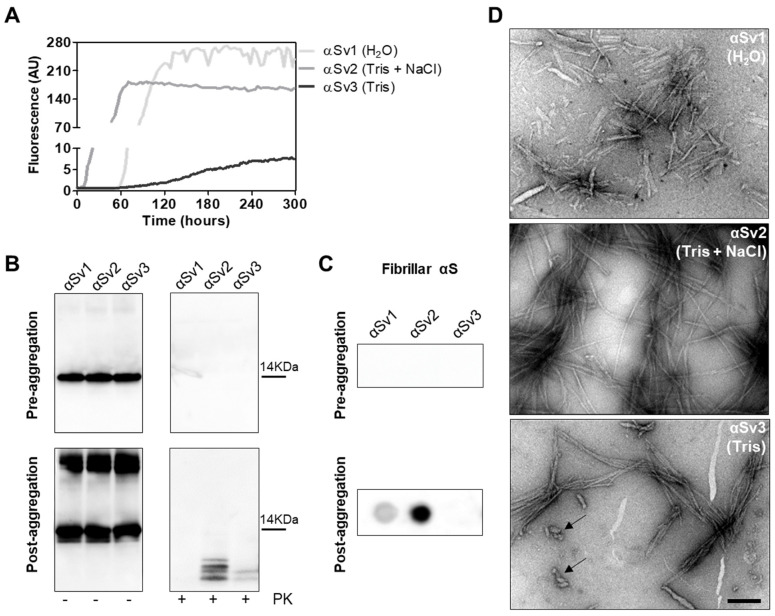
(**A**) Kinetics of αSv1, αSv2 and αSv3 aggregation. Rec-αSyn was induced to aggregate in H_2_O (αSv1, light grey line), 5 mM Tris and 100 mM NaCl (αSv2, dark grey line) or 5 mM Tris (αSv3, black line), under continuous shaking, and the aggregation was monitored with the use of ThT. Curves represented in the graph were obtained by plotting the average fluorescence intensities of each sample against time. (**B**) Western blot analysis of untreated or PK digested αSv1, αSv2 and αSv3. Before aggregation, the rec-αSyn was completely digested, regardless of the reaction buffer. After aggregation, αSv1 was completely digested by PK, αSv2 showed four PK-resistant bands while αSv3 showed two PK-resistant bands. Samples were immunoblotted with the AS08 358 antibody. Number on the right indicates the molecular weight. (**C**) Dot blot analysis of αSv1, αSv2 and αSv3 using MJFR antibody. As expected, before aggregation, MJFR did not recognize αSyn fibrils. After aggregation, αSv1 and αSv2 fibrils were recognized by MJFR (although with different affinity) while αSv3 fibrils did not. (**D**) TEM analysis of αSv1, αSv2 and αSv3. TEM analysis showed that αSv1, αSv2 and αSv3 possessed different morphological features. Arrows indicate amorphous material found together with αSv3 fibrils. Scale bar = 200 nm.

To better investigate whether the αSyn^D^ strains responsible for MSA and PD might have influenced the distinctive biochemical properties of RQ-MSA and RQ-PD, we decided to generate three different artificial αSyn amyloid fibrils, named αSv1, αSv2 and αSv3. These aggregates were produced starting from rec-αSyn that was incubated in three different buffers without ThT (Table 1). To monitor the formation of αSyn fibrils in each experimental condition, a group of samples was supplemented with ThT (Figure 4A). At the end of the aggregation (monitored by ThT), samples were extensively characterized from a biochemical and morphological point of view. In particular, αSv1 fibrils mostly aggregated side-by-side, were sensitive to PK treatment and barely recognized by the MJFR antibody (Figure 4B–D). In contrast, αSv2 fibrils were generally longer than those of αSv1 and did not aggregate side-by-side but formed a net-like structure. In addition, over-twists were detectable at regular intervals in most of the fibrils. These aggregates were partially resistant to proteolytic digestion and four PK-resistant bands (migrating between 3 and 14 kDa) were detected by Wb. The MJFR antibody well-interacted with the sample (Figure 4B–D). Finally, αSv3 fibrils aggregated either side-by-side or by forming a net-like structure and many of them showed the presence of over-twists. We have also observed the presence of some amorphous material that was not found in the other samples (see arrows in Figure 4D). αSv3 was less resistant to enzymatic digestion than αSv2 and two PK-resistant bands were observed at Wb, always migrating between 3 and 14 kDa. In this case, the MJFR antibody did not interact with the fibrils (Figure 4B–D). The differences in PK resistance properties, biochemical profiles, affinity toward the MJFR antibody, and TEM findings of αSv1, αSv2 and αSv3 demonstrated that we efficiently generated distinct artificial aggregates of αSyn (Figure 4B–D, Table 1). Finally, αSv1, αSv2 and αSv3 were incubated with eight fluorescent probes which differently interacted with the aggregates, hence confirming that they acquired distinct morphological properties (Supplementary Figure S1).

### 3.4. αSyn_RT-QuIC Analysis of Recombinant α-Synuclein Aggregates and Characterization of Final Reaction Products

**Figure 5:**
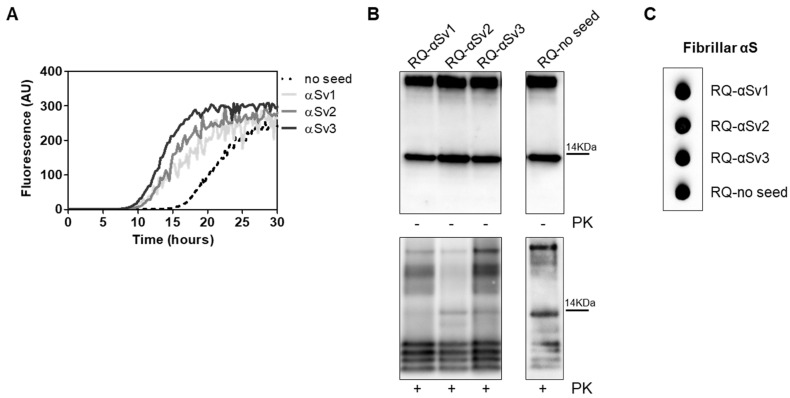
(**A**) αSyn_RT-QuIC analysis of αSv1, αSv2 and αSv3. Minute amounts (1.5 ag) of αSv1, αSv2 and αSv3 were tested in αSyn_RT-QuIC and induced an efficient seeding activity, with respect to the control (no seed). Curves represented in the graph were obtained by plotting the average fluorescence intensities of each sample against time. (**B**) Western blot analysis of untreated or PK digested αSyn_RT-QuIC products, collected at 30 h. RQ-αSv1, RQ-αSv2, RQ-αSv3 and RQ-no seed showed similar signal intensities before digestion. After PK treatment, all samples showed similar resistance to digestion and comparable biochemical profiles (AS08 358 antibody). Number on the right indicates the molecular weight. (**C**) Dot blot analysis of αSyn_RT-QuIC products, collected at 30 h. Dot blot analysis performed using the MJFR antibody showed the presence of a strong signal in all samples.

αSv1, αSv2 and αSv3 were subjected to αSyn_RT-QuIC analysis to test whether they could transmit their specific morphological features to the reaction substrate. All aggregates induced a seeding activity (Figure 5A), even when tested at very low dilutions (attograms) (Supplementary Figure S2). This finding showed that traces of αSv1, αSv2 and αSv3 triggered rec-αSyn aggregation with an efficiency similar to that of OM samples. However, although the αSv1, αSv2 and αSv3 were characterized by distinctive features, their αSyn_RT-QuIC reaction products (named RQ-αSv1, RQ-αSv2 and RQ-αSv3, respectively) did not retain these properties and showed similar PK resistant bands at Wb. Notably, aggregates were also found in RQ-no seed and their biochemical properties were comparable to those of RQ-αSv1, RQ-αSv2 and RQ-αSv3 (Figure 5B). In particular, we observed four bands in each sample, migrating between 3 and 14 kDa. In addition, MJFR antibody recognized with similar affinity RQ-αSv1, RQ-αSv2, RQ-αSv3 and RQ-no seed (Figure 5C). Therefore, while OM-MSA and OM-PD samples were able to generate αSyn_RT-QuIC products with distinctive properties, the in vitro generated αSv1, αSv2 and αSv3, although characterized by different morphological features, did not. For this reason, we hypothesized that only the αSyn^D^ strains present in OM samples and likely other tissue factors (e.g., microbiota [43]) could markedly influence the misfolding process of rec-αSyn and the biochemical and morphological features of the RT-QuIC products. Hence, αSv1, αSv2 and αSv3 produced from rec-αSyn, lacked peculiar features which are present in the natural α-synuclein strains (e.g., phosphorylation) that could play a role in this process.

### 3.5. Inflammatory Profile of SH-SY5Y Cells Exposed to αSv1, αSv2, αSv3 and Their αSyn_RT-QuIC Reaction Products

**Figure 6:**
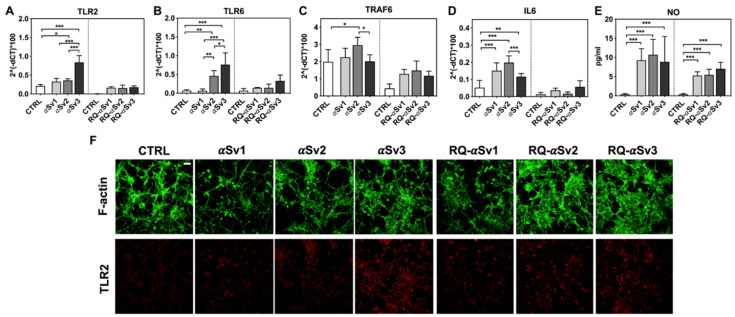
Analysis of inflammatory molecules in differentiated SH-SY5Y cells stimulated with αSv1, αSv2, αSv3 and their αSyn_RT-QuIC reaction products (RQ-αSv1, RQ-αSv2, and RQ-αSv3). RT-qPCR analysis of TLR2 (**A**), TLR6 (**B**), TRAF6 (**C**), IL6 (**D**) showed an upregulation of the inflammatory mediators transcript levels in cells stimulated with αSv1, αSv2, αSv3 but not in those stimulated with RQ-αSv1, RQ-αSv2, and RQ-αSv3. (**E**) NO quantification in the supernatants of cells stimulated with αSv1, αSv2, αSv3, RQ-αSv1, RQ-αSv2, and RQ-αSv3 and related control buffers. A significant increase in NO release was found only in αSv1, αSv2, αSv3, RQ-αSv1, RQ-αSv2, and RQ-αSv3 stimulated cells. Values are expressed as mean ± SD. Statistical significance was assessed by one-way ANOVA test with Dunnett’s multiple comparison test. Corrected *p*-values are reported in the results section. * *p* ≤ 0.05, ** *p* ≤ 0.01, *** *p* ≤ 0.001. (**F**) Immunofluorescence analysis of TLR2 expression (red) in SH-SY5Y treated cells or in control condition showing an increase in protein expression in all SH-SY5Y stimulated cells that was more intense in those challenged with αSv3. Counterstaining with Alexa-Fluor 488-Phalloidin (green) was performed to monitor the cytoskeleton structure. Bar scale = 25 µm.

**Figure 7:**
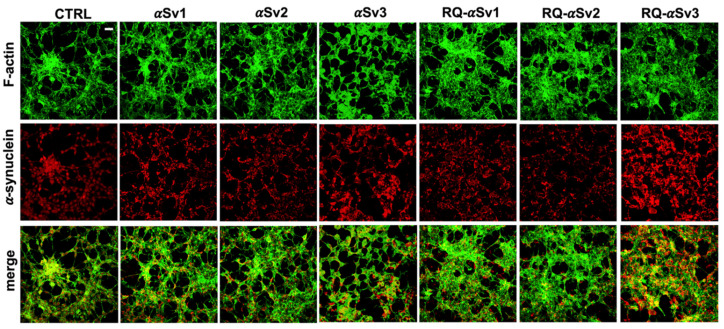
Intracellular αSyn aggregates in SH-SY5Y cells stimulated with αSv1, αSv2, αSv3 and their αSyn_RT-QuIC reaction products (RQ-αSv1, RQ-αSv2, and RQ-αSv3). SH-SY5Y cells, stimulated with αSv1, αSv2, αSv3, RQ-αSv1, RQ-αSv2, RQ-αSv3 (2.5 μM for 24 h) and control (CTRL), were stained for αSyn (red) to visualize the cellular aggregates. With respect to CTRL, all stimulated SH-SY5Y cells showed clusters of αSyn aggregates that were more abundant in those challenged with both αSv3 and RQ-αSv3. Cells were counterstained with Alexa-Fluor 488-Phalloidin (green) to highlight the thin neurite-like cytoplasmic structures (cell-to-cell contacts) denoted by F-actin filaments. The cytoskeleton of the cells was preserved, regardless of the presence of αSyn aggregates. Scale bar = 25 µm.

As RQ-MSA and RQ-PD induced distinct inflammatory responses in neuron-like SH-SY5Y cells, we then evaluated the inflammatory effects of αSv1, αSv2, αSv3 and their αSyn_RT-QuIC reaction products in the same cellular model. This experiment was aimed at demonstrating a correlation between the biochemical/morphological features of the αSyn aggregates and the inflammatory responses observed in cells. In particular, we performed RT-qPCR analyses of selected inflammatory mRNAs in cells exposed to αSv1, αSv2, and αSv3 or to their reaction buffers (devoid of rec-αSyn) as controls (Figure 6). The analysis showed differential expression of TLR2, TLR6, TRAF6 and IL6 transcripts. Since SH-SY5Y cells stimulated with all the three control buffers showed analogous responses, the H_2_O condition was chosen as representative control (CTRL). In particular, the expression of TLR2 was significantly higher in cells exposed to αSv3 than those exposed to αSv1 and αSv2 (αSv3 vs. αSv2: *p* < 0.0001; αSv3 vs. αSv1: *p* < 0.0001) or control (αSv3 vs. CTRL: *p* < 0.0001) (Figure 6A) and this increase was also confirmed at protein level by confocal microscopy analysis (Figure 6F, red color); the expression of TLR6 was found higher in cells exposed to αSv2 and αSv3 than those exposed to αSv1 or CTRL. These differences were statistically significant (αSv1 vs. αSv2: *p* = 0.002; αSv1 vs. αSv3: *p* < 0.0001; αSv2 vs. αSv3: *p* = 0.03; αSv2 vs. CTRL: *p* = 0.003; αSv3 vs. CTRL: *p* < 0.0001) (Figure 6B); with respect to the cells challenged with control buffers, the expression of TRAF6 was significantly higher only in those exposed to αSv2 and a statistically significant difference was also found between αSv2 and αSv3 stimulated cells (*p* = 0.017) or CTRL (*p* = 0.013) (Figure 6C); finally, a statistically significant increase of IL6 was observed in all stimulated cells compared to controls (αSv1 vs. CTRL: *p* = 0.0002; αSv2 vs. CTRL: *p* < 0.0001; αSv3 vs. CTRL: *p* = 0.007). Among challenged cells, a statistically significant difference was observed only between αSv2 and αSv3 (*p* = 0.0007) (Figure 6D). We have then analyzed a panel of pro-inflammatory mediators via multiparametric assays in the supernatants of stimulated cells and found a general upregulation of IFNγ, IL1β, IL1ra, IL2, IL4, IL6 IL9, IL15, IL17, IP10 and TNFα with respect to CTRL (Supplementary Figure S3). Finally, we have assessed the production of NO (Figure 6E) which was found increased with respect to the controls but not significantly different among αSv1, αSv2, αSv3 (αSv1 vs. CTRL: *p* = 0.0002; αSv2 vs. CTRL: *p* < 0.0001; αSv3 vs. CTRL: *p* = 0.0004). No alterations in the structure of the cytoskeleton of stimulated cells were detected, as confirmed by F-actin staining (Figure 6F, green color). Finally, accumulation of α-synuclein was observed in all stimulated cells, and the highest signal intensity was observed in those challenged with αSv3 (Figure 7, red color). Regarding the cells stimulated with RQ-αSv1, RQ-αSv2, RQ-αSv3, respectively, we have found analogous expression levels of inflammatory mediators, including TLR2, TLR6, TRAF6, and IL6 that were higher than those of cells stimulated with CTRL (Figure 6). However, these differences did not reach a statistical significance. In contrast, we have observed a significantly higher production of NO in cells stimulated with RQ-αSv1, RQ-αSv2, and RQ-αSv3 compared to the control (RQ-αSv1 vs. CTRL: *p* < 0.0001; RQ-αSv2 vs. CTRL: *p* < 0.0001; RQ-αSv3 vs. CTRL: *p* < 0.0001). However, although we did not observe significantly different inflammatory responses in cells stimulated with RQ-αSv1, RQ-αSv2, and RQ-αSv3, immunofluorescence analysis revealed that cells stimulated with RQ-αSv3 showed higher amount of αSyn aggregates than the others. Remarkably, higher accumulation of αSyn was also observed in cells stimulated with αSv3 (Figure 7, red color). Thus, even if the biochemical profiles of αSv3 and RQ-αSv3 differed between each other, both samples showed stronger seeding activity for αSyn when challenged in SH-SY5Y cells.

### 3.6. αSyn_RT-QuIC Analysis of Lysates from Cells Stimulated with αSv1, αSv2, αSv3 and RQ-MSA and RQ-PD

**Figure 8:**
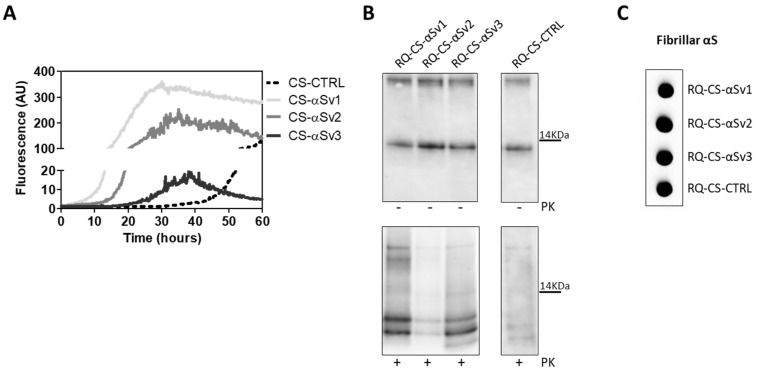
(**A**) αSyn_RT-QuIC analysis of CS-αSv1, CS-αSv2 and CS-αSv3. All samples triggered a seeding activity when tested by αSyn_RT-QuIC. Curves represented in the graph were obtained by plotting the average fluorescence intensities of each sample against time. (**B**) Western blot analysis of untreated or PK digested αSyn_RT-QuIC products, collected at 60 h. Undigested samples showed the same biochemical profiles. PK treatment of αSyn_RT-QuIC products (RQ-CS-αSv1, RQ-CS-αSv2 and RQ-CS-αSv3 and RQ-CS-CTRL) showed that the samples were characterized by similar biochemical profiles, resulting in the formation of three PK resistant bands migrating between 3 and 14 kDa. Membranes were immunoblotted with the AS08 358 antibody. Number on the right indicates the molecular weight. (**C**) Dot blot analysis of αSyn_RT-QuIC products, collected at 60 h. Dot blot analysis performed using the MJFR antibody showed the presence of a strong signal in all the samples.

The lack of biochemical differences among RQ-αSv1, RQ-αSv2, RQ-αSv3 could be due to the artificial nature of αSv1, αSv2, and αSv3, as previously mentioned. For this reason, the αSyn aggregates formed in SH-SY5Y cells stimulated with αSv1, αSv2, αSv3 (CS-αSv1, CS-αSv2, and CS-αSv3, respectively) were subjected to αSyn_RT-QuIC analysis considering that their composition might be more similar to that of the natural αSyn^D^ strains (e.g., presence of phosphorylation). Indeed, it has already been shown that the stimulation of SH-SY5Y cells with PFFs induced intracellular accumulation of phosphorylated αSyn, which is similar to that aggregating in the brains of patients with α-synucleinopathies [44]. All samples induced an efficient seeding activity with respect to the control (CS-CTRL). Notably, the kinetics of rec-αSyn aggregation stimulated by αSv3 (Figure 4A) and CS-αSv3 (Figure 8A) were found to be less efficient compared to the other samples. The final αSyn_RT-QuIC products generated by CS-αSv1, CS-αSv2, CS-αSv3 and CS-CTRL (named RQ-CS-αSv1, RQ-CS-αSv2, RQ-CS-αSv3 and RQ-CS-CTRL, respectively) were subjected to Wb analysis after PK digestion and showed the presence of three PK resistant bands, thus confirming the lack of biochemical differences among specimens (Figure 8B). Dot blot analysis revealed that the MJFR antibody was able to bind all the aggregates with similar affinity, sustaining that they all possessed similar morphological properties (Figure 8C).

**Figure 9:**
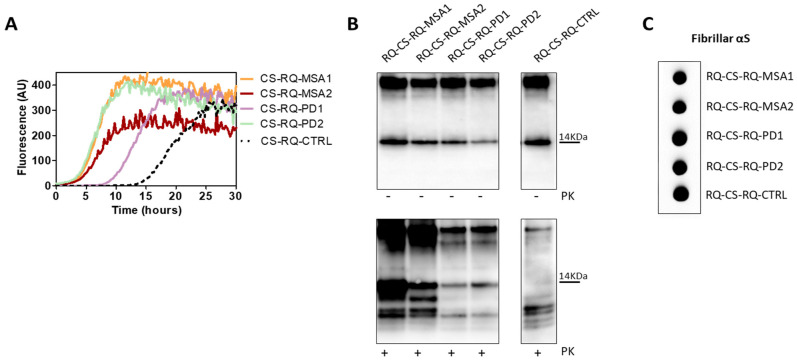
(**A**) αSyn_RT-QuIC analysis of CS-RQ-MSA and CS-RQ-PD. All samples were tested by αSyn_RT-QuIC and induced an efficient seeding activity with respect to the control (CS-RQ-CTRL). Curves represented in the graph were obtained by plotting the average fluorescence intensities of each sample against time. (**B**) Western blot analysis of untreated or PK digested αSyn_RT-QuIC products, collected at 30 h. Undigested samples showed the same biochemical profiles. Western blot analysis of PK digested RQ-CS-RQ-MSA1 and RQ-CS-RQ-MSA2 showed higher resistance to proteolytic digestion than RQ-CS-RQ-PD1 and RQ-CS-RQ-PD2. Membranes were immunoblotted with the AS08 358 antibody. Number on the right indicates the molecular weight. (**C**) Dot blot analysis of αSyn_RT-QuIC products, collected at 30 h. Dot blot analysis performed using the MJFR antibody showed the presence of a strong signal in all samples.

These findings demonstrate that even the αSyn aggregates produced in cells were not able to imprint distinctive features to the αSyn_RT-QuIC products. However, we have noticed a difference between the biochemical profiles of RQ-αSv1, RQ-αSv2, RQ-αSv3 (Figure 4B) and those of RQ-CS-αSv1, RQ-CS-αSv2 and RQ-CS-αSv3 (Figure 8B). Finally, we have decided to test whether the lysates of cells stimulated with RQ-MSA and RQ-PD were still able to trigger rec-αSyn aggregation by αSyn_RT-QuIC and investigate their biochemical properties. As control, we have used cells stimulated with unseeded αSyn_RT-QuIC reaction mix. These samples were named CS-RQ-MSA, CS-RQ-PD and CS-RQ-CTRL, respectively and, except the control, efficiently seeded the aggregation of rec-αSyn (Figure 9A). Surprisingly, the biochemical profiles of the αSyn_RT-QuIC products were similar among each-others but those generated by CS-RQ-MSA were more resistant than those generated by CS-RQ-PD (Figure 9B). Such a different sensitivity toward PK digestion of MSA and PD generated samples was also observed in RQ-MSA and RQ-PD (Figure 1B). However, the biochemical profiles of resulting αSyn_RT-QuIC products obtained from both experimental conditions have changed, suggesting that the passage in cells might have further modified the αSyn^D^ strain properties of MSA and PD. Dot blot analysis revealed that the MJFR antibody was able to bind all the aggregates with similar affinity (Figure 9C).

## 4. Discussions

MSA and PD are neurodegenerative diseases caused by different strains of αSyn^D^. Variations in the aberrant structure of αSyn^D^ are believed to give the protein disease-specific pathological features, including the capability to activate distinct inflammatory pathways. In 2019, we have shown that OM samples collected from patients with MSA and PD were able to seed αSyn_RT-QuIC reaction, leading to the formation of distinct αSyn aggregates, named RQ-MSA and RQ-PD. Notably, RQ-MSA samples were more resistant to PK digestion than RQ-PD.

In this work, we have demonstrated that RQ-MSA and RQ-PD induced different levels of inflammatory responses when challenged in neuronal-like differentiated SH-SY5Y cells. Other than confirming their ability to seed the aggregation of endogenous αSyn, RQ-MSA elicited a significant increase in the transcription levels of several inflammatory mediators, including TLR2, TRAF6, IL6, NLRP3, SOD2 than those activated by RQ-PD and controls. Notably, the level of transcription factors activated in cells stimulated with RQ-PD was slightly higher than that of cells stimulated with control, but this difference was not statistically significant. This indicates that RQ-PD possesses less inflammatory features than RQ-MSA, even though both samples induced a significant increase of NO release in stimulated cells compared to controls.

These findings suggested the existence of a link between the morphology of the aggregates and their inflammatory properties. Since the OM samples contain several components, besides αSyn^D^, that could have influenced the misfolding of rec-αSyn, we have decided to evaluate to what extent the aberrant structures of αSyn^D^ could have impacted this process. Hence, we have produced three different aggregates of αSyn (αSv1, αSv2, αSv3) starting from the same rec-αSyn. The aim of this experiment was to generate, in a controlled environment, artificial αSyn seeds, resembling to some extent the αSyn^D^ strains present in OM, and test their behavior by αSyn_RT-QuIC without the presence of specific tissue factors. Although capable to efficiently seed rec-αSyn aggregation, αSv1, αSv2, and αSv3 did not transmit their seed-specific properties to the reaction products which showed comparable biochemical properties, instead. Probably, our experimental setting was too artificial to properly recapitulate the phenomenon of the seeding effect exerted by αSyn^D^ in αSyn_RT-QuIC.

However, when used to stimulate SH-SY5Y cells, αSv1, αSv2, and αSv3 acted on different activators of inflammatory pathways, thus strengthening the existence of a correlation between morphological and inflammatory properties of αSyn fibrils. All stimulated cells showed aggregates of endogenous αSyn that could be more similar to the αSyn^D^ present in OM samples with respect to the artificial αSv1, αSv2, and αSv3. For this reason, we have lysed the cells and tested the lysates by αSyn_RT-QuIC to verify whether they could seed the reaction and generate final products eventually showing distinctive biochemical properties. Although the cell-derived aggregates were able to seed the reaction, resulting αSyn fibrils (named RQ-CS-αSv1, RQ-CS-αSv2, and RQ-CS-αSv3) showed similar biochemical properties. Notably, by comparing the biochemical profiles of the αSyn_RT-QuIC products generated by αSv1, αSv2, and αSv3 (RQ-αSv1, RQ-αSv2, RQ-αSv3) with those obtained from cells stimulated with them (RQ-CS-αSv1, RQ-CS-αSv2, and RQ-CS-αSv3), we have observed that the number of PK resistant αSyn bands were different. In particular, four bands were found in the first case and three bands in the second one. This finding indicates that the aggregates generated in cells, hence in a more physiological environment, might have acquired conformations that were slightly different from that of αSv1, αSv2, and αSv3. Therefore, the properties of these αSyn aggregates (e.g., presence of post-translational modifications), the existence of specific cellular components (e.g., different microenvironments), or both, might have influenced the biochemical properties of the final αSyn_RT-QuIC products further sustaining that they could depend on several factors, not only on the structure and composition of the original αSyn seeds.

Unfortunately, we did not have enough OM samples for a direct stimulation of the cells that were stimulated with RQ-MSA and RQ-PD, instead. Cells were then lysed and lysates subjected to αSyn_RT-QuIC analysis. All samples were able to seed the aggregation of rec-αSyn and final reaction products (RQ-CS-RQ-MSA and RQ-CS-RQ-PD) did not show distinct biochemical profiles but, surprisingly, those generated from RQ-MSA were again more resistant to PK digestion than those generated by RQ-PD. Also in this case, the passage in cells has partially altered the biochemical features of RQ-MSA and RQ-PD. For this reason, we can assume that passaging of αSyn aggregates in cells might alter their original features, regardless of the origins (artificial vs. natural).

Our study, although performed on a limited number of samples, showed that there might be an association between the aberrant conformations of the αSyn aggregates and the inflammatory responses that they are capable to induce in SH-SY5Y stimulated cells. It is important to verify whether the inflammatory pathways altered in RQ-MSA and RQ-PD stimulated cells might resemble those eventually altered by αSyn^D^ responsible for MSA and PD. If this was the case, stimulation of cells with OM generated αSyn_RT-QuIC aggregates can be exploited to study the molecular events associated with αSyn misfolding and aggregation in vitro and eventually identify novel disease-specific therapeutic targets. Finally, we have observed that not only the structure of these aggregates, but also other environmental factors might play a role in modulating the final properties of the αSyn_RT-QuIC reaction products. Hence, the discovery of specific environmental modulators involved in αSyn^D^ misfolding in MSA and PD might further help to plan innovative targeted therapies.

## Figures and Tables

**Table 1:** Summary of the characteristics of recombinant α-synuclein variants.

αSyn Variant	Aggregation Buffer	Biochemical Properties of the Fibrils	Morphological Properties of the Fibrils		
			Length	Over-Twists	Note
αSv1	H_2_O	Completely digested by PK	Short	No	Arranged side-by-side
αSv2	5 mM Tris + 100 mM NaCl	Partially resistant to digestion (4 PK resistant bands detected)	Long	Yes, although few fibrils without over-twists were also found	Arranged in a net-like structure
αSv3	5 mM Tris	Partially resistant to digestion (2 PK resistant bands detected)	Mainly short	Yes, although several fibrils without over-twists were also found	Arranged either side-by-side or in a net-like structure. Presence of amorphous material

## Data Availability

All data relevant to the study are included in the article or uploaded as Supplementary Materials.

## Supplementary Materials

The following supporting information can be downloaded at: https://www.mdpi.com/article/10.3390/cells11010087/s1. Figure S1: Dye-binding assay of rec-αSyn aggregates, Figure S2: αSyn_RT-QuIC analysis of serial dilutions of αSv1, αSv2 and αSv3, Figure S3: Multiparametric analysis of inflammatory mediator production from stimulated SH-SY5Y.
