# Supplementary material for: The Alpha-Synuclein RT-QuIC Products Generated by the Olfactory Mucosa of Patients with Parkinson’s Disease and Multiple System Atrophy Induce Inflammatory Responses in SH-SY5Y Cells
Source: Cells. 2021 Dec 28;11(1):87. doi: 10.3390/cells11010087 (PMC8750063; doi:10.3390/cells11010087)
Supplement: Supplementary file 1 [file cells-11-00087-s001.zip › cells-1443263-supplementary.pdf]

Supplementary Informations

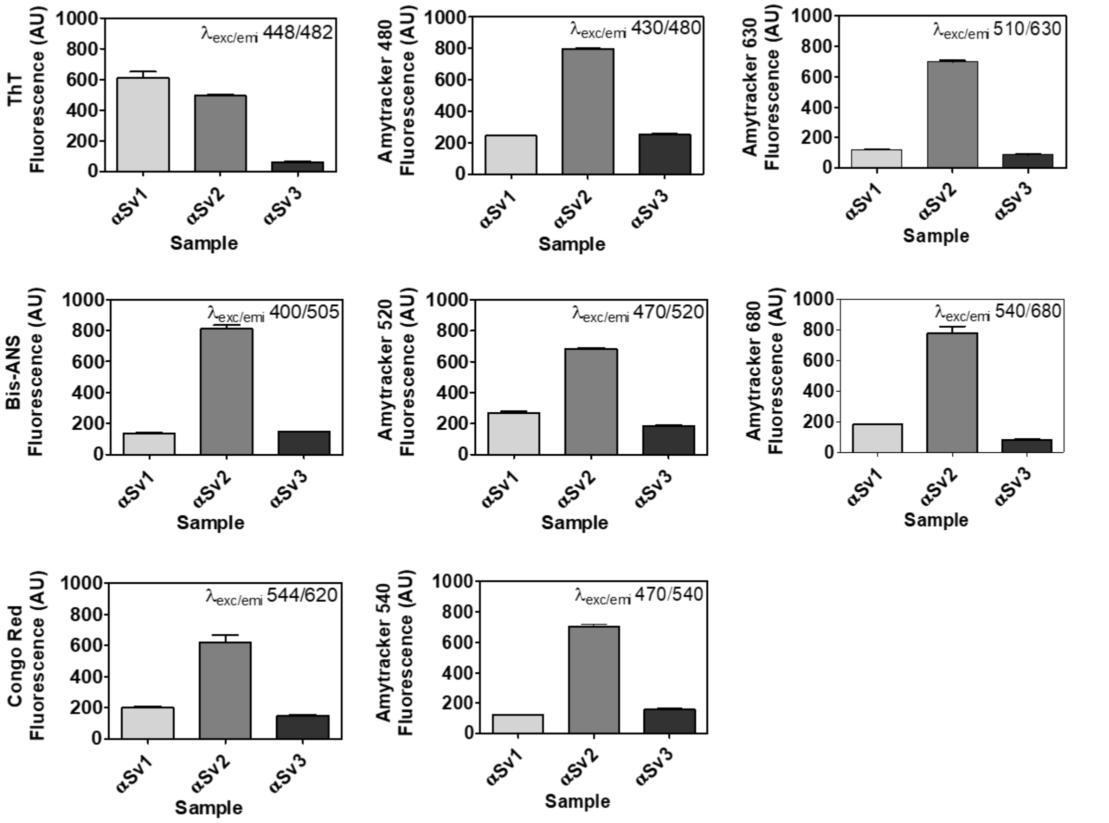

Supplementary Figure S1. Dye-binding assay of rec-αSyn aggregates. Once generated, αSv1, αSv2 and αSv3 were incubated with ThT, Bis-ANS, Congo red, Amytracker 480, Amytracker 520, Amytracker 540, Amytracker 630, Amytracker 680. αSv1 interacted with higer efficiency with ThT than αSv2 and αSv3. αSv2 interacted with higher efficiency with all the other fluorophores while αSv3 weakly interacted with all the fluorescent probes.

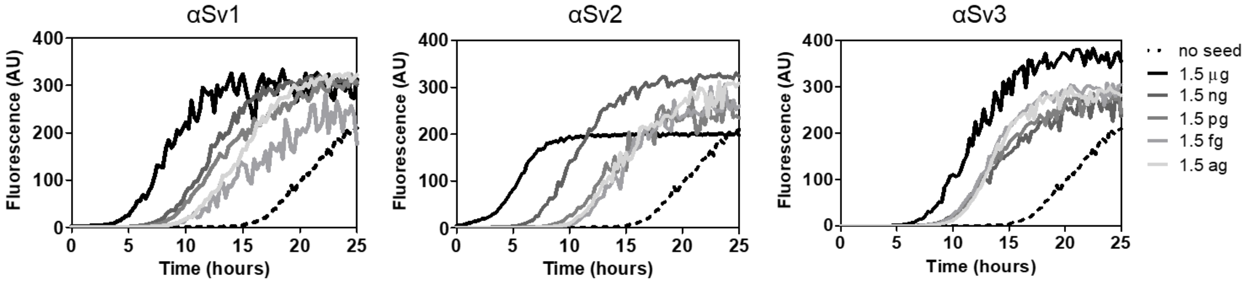

Supplementary Figure S2. αSyn_RT-QuIC analysis of serial dilutions of αSv1, αSv2 and αSv3. All dilutions were able to induced an efficient seeding activity by αSyn_RT-QuIC. Curves represented in the graph were obtained by plotting the average fluorescence intensities of each sample against time.

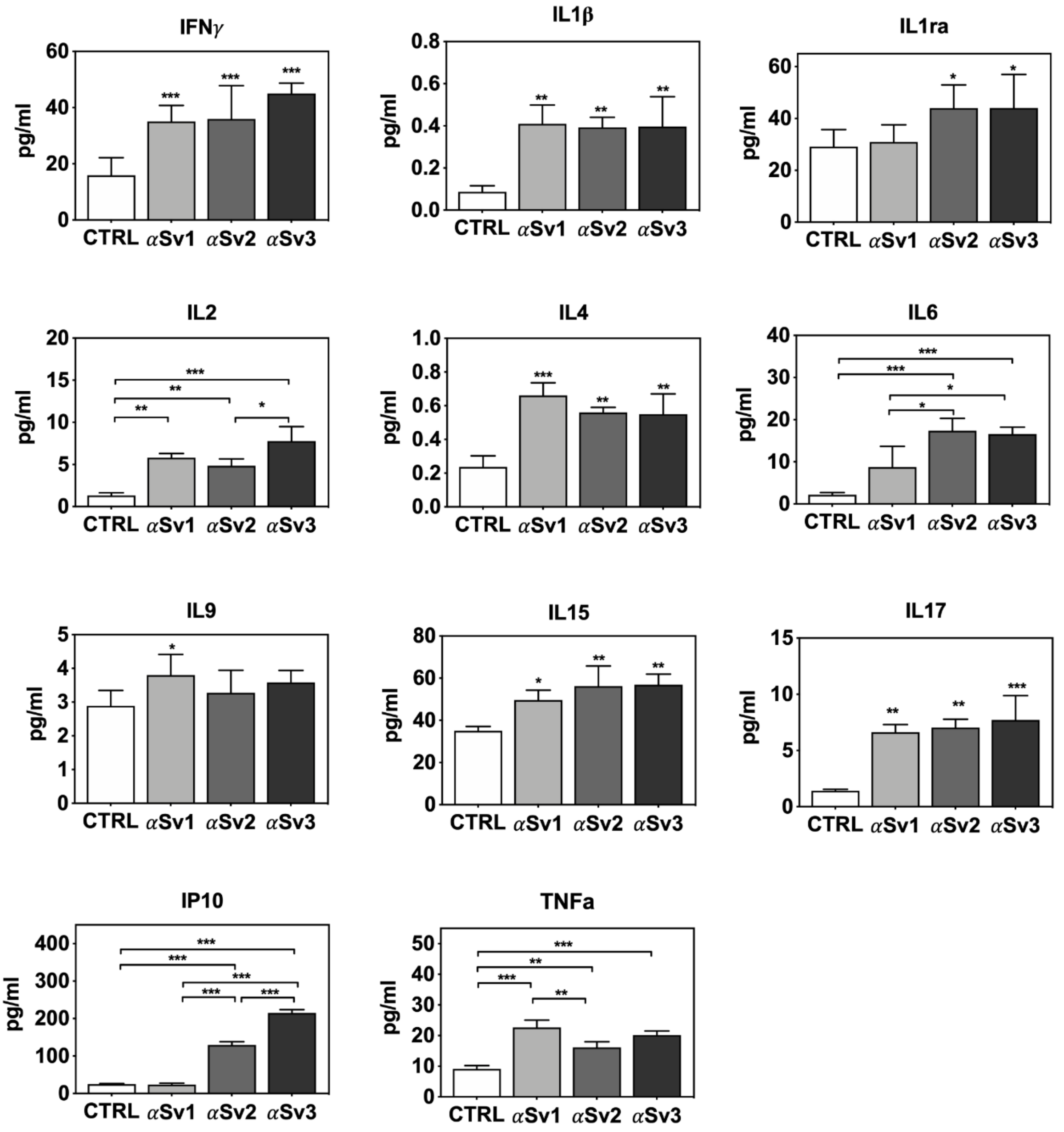

Supplementary Figure S3. Multiparametric analysis of inflammatory mediator production from stimulated SH-SY5Y. Immune mediator quantification in the supernatants of SH-SY5Y incubated with αSv1, αSv2, αSv3 (2.5 μM for 24 hours), and control buffer medium. Among the 27 molecules analyzed, the serum concentration levels of 11 cytokines was found statistically significant: IFNγ (αSv1 vs control: p=0.0008; αSv2 vs control: p=0.0005; αSv3 vs control: p<0.0001); IL1α(βSv1 vs control: p=0.0052; αSv2 vs control: p=0.0070; αSv3 vs control: p=0.0066); ILra (αSv2 vs control: p=0.04; aSv3 vs control: p=0.03); IL2 (αSv1 vs control: p=0.0015; αSv2 vs control: p=0.0067; αSv3 vs control: p=0.0001; αSv2 vs αSv3: p=0.02); IL4 (αSv1 vs control: p=0.0005; αSv2 vs control: p=0.0028; αSv3 vs control: p=0.0034); IL6 (αSv2 vs control: p=0.0007; αSv3 vs control: p=0.001; αSv1 vs αSv2: p=0.02; αSv1 vs αSv3: p=0.03); IL9 (αSv1 vs control: p=0.02); IL15 (αSv1 vs control: p=0.04; αSv2 vs control: p=0.006; αSv3 vs control: p=0.0051); IL17 (αSv1 vs control: p=0.0017; αSv2 vs control: p=0.0011; αSv3 vs control: p=0.0005); IP10 (αSv2 vs control: p<0.0001; α Sv3 vs control: p<0.0001; αSv1 vs αSv2: p<0.0001; αSv1 vs αSv3: p<0.0001; αSv2 vs αSv3: p<0.0001); TNFα(αSv1 vs control: p<0.0001; αSv2 vs control: p=0.0028; αSv3 vs control: p=0.0002; αSv1 vs αSv2: p=0.0052). Statistical significance was assessed by one-way ANOVA test with Dunnett's multiple comparison test. * p ≤ 0.05, ** p ≤ 0.01, *** p ≤ 0.001.
